# Immunomodulation by the *Pseudomonas syringae* HopZ Type III Effector Family in Arabidopsis

**DOI:** 10.1371/journal.pone.0116152

**Published:** 2014-12-29

**Authors:** Jennifer D. Lewis, Mike Wilton, G . Adam Mott, Wenwan Lu, Jana A. Hassan, David S. Guttman, Darrell Desveaux

**Affiliations:** 1 Department of Cell and Systems Biology, University of Toronto, Toronto, Ontario, Canada; 2 Plant Gene Expression Center, United States Department of Agriculture, Albany, California, United States of America; 3 Department of Plant and Microbial Biology, University of California, Berkeley, California, United States of America; 4 Centre for the Analysis of Genome Evolution and Function, University of Toronto, Toronto, Ontario, Canada; Indiana University, United States of America

## Abstract

*Pseudomonas syringae* employs a type III secretion system to inject 20–30 different type III effector (T3SE) proteins into plant host cells. A major role of T3SEs is to suppress plant immune responses and promote bacterial infection. The YopJ/HopZ acetyltransferases are a superfamily of T3SEs found in both plant and animal pathogenic bacteria. In *P. syringae*, this superfamily includes the evolutionarily diverse HopZ1, HopZ2 and HopZ3 alleles. To investigate the roles of the HopZ family in immunomodulation, we generated dexamethasone-inducible T3SE transgenic lines of Arabidopsis for HopZ family members and characterized them for immune suppression phenotypes. We show that all of the HopZ family members can actively suppress various facets of Arabidopsis immunity in a catalytic residue-dependent manner. HopZ family members can differentially suppress the activation of mitogen-activated protein (MAP) kinase cascades or the production of reactive oxygen species, whereas all members can promote the growth of non-virulent *P. syringae*. Localization studies show that four of the HopZ family members containing predicted myristoylation sites are localized to the vicinity of the plasma membrane while HopZ3 which lacks the myristoylation site is at least partially nuclear localized, suggesting diversification of immunosuppressive mechanisms. Overall, we demonstrate that despite significant evolutionary diversification, all HopZ family members can suppress immunity in Arabidopsis.

## Introduction

Plants initiate immune responses after perception of microbe-associated molecular patterns (MAMPs) from diverse pathogens by membrane associated pattern recognition receptors (PRRs) [1]. This PRR-triggered immunity (PTI) results in the production of reactive oxygen species (ROS) [2], activation of mitogen-activated protein kinase (MAPK) cascades [3] and transcriptional reprogramming [4]. These events result in strengthening of the cell wall by callose deposition [5], and restriction of bacterial growth [4].

Over evolutionary time, pathogens have developed effector proteins that suppress PTI and allow the pathogen to cause disease. *P. syringae* employs the type III secretion system (T3SS) as a primary virulence strategy to suppress PTI. The T3SS is a needle-like syringe that delivers type III secreted effector proteins (T3SEs) into the plant cell, where they can disrupt immune signaling pathways [6]. In a classic example of the plant-pathogen arms race, plants have evolved nucleotide binding leucine-rich repeat resistance proteins (NLRs) that recognize specific T3SEs, resulting in effector-triggered immunity (ETI) [7]. ETI displays characteristics of an accelerated and amplified PTI response that commonly culminates in a programmed cell death hypersensitive response (HR) [8].

T3SEs have evolved to disrupt various aspects of both PTI and ETI, in order to restore bacterial virulence. Specific T3SEs have been shown to target MAPK signaling cascades, PRR complexes, PTI transcriptional regulators, or ETI signaling components to suppress both branches of plant immunity [9], [10], [11]. In addition, effectors can target more general plant systems such as the proteasome, the cytoskeleton, or the secretion pathway to indirectly alter plant immunity [9], [10].

The YopJ/AvrRxv/HopZ family of T3SEs is evolutionary diverse and found in both animal and plant bacterial pathogens [12], [13]. The HopZ family members are found in *P. syringae* strains infecting a range of hosts: HopZ1a*_Psy_*_A2_ (hereafter HopZ1a) from a pear-infecting strain; HopZ1b*_Pgy_*_UnB647_ (hereafter HopZ1b) from a kidney bean-infecting strain; HopZ1c*_Pma_*_ES4326_ (hereafter HopZ1c) from a radish-infecting strain; HopZ2*_Ppi_*_895A_ (hereafter HopZ2) from a pea-infecting strain; and HopZ3*_Psy_*_B728a_ (hereafter HopZ3) from a snap bean-infecting strain. In *P. syringae*, HopZ1a, HopZ1b and HopZ1c diversified by pathoadaptation due to selective pressures from host recognition, while HopZ2 and HopZ3 were acquired by horizontal gene transfer from other plant pathogens [12]. Recently, a novel member of the HopZ family, HopZ4*_Pla_*_107_ from a cucumber-infecting strain, was described [14] (see Discussion). Functional diversification of this family has been characterized quite extensively in Arabidopsis. HopZ1a is the only HopZ family member to give a strong ETI response in Arabidopsis, and this has been shown to be mediated by the ZAR1 NLR protein and the ZED1 pseudokinase [15], [16], [17]. HopZ1b causes a weak HR-like response in Arabidopsis that is independent of ZAR1 [15], [16]. HopZ2 is able to enhance the apoplastic growth of *P. syringae*, whereas HopZ3 contributes to epiphytic growth of *P. syringae* on Arabidopsis [15], [16], [18]. HopZ1c has yet to be ascribed a function *in planta*.

All members of the HopZ family possess a catalytic triad (C/H/D) characteristic of enzymes such as proteases and acetyltransferases. Acetyltransferase activity has been demonstrated for HopZ1a, and the catalytic triad cysteine is required for HopZ avirulence and virulence functions [15], [16], [19], [20]. In addition, all members of the HopZ family except HopZ3 possess a predicted myristoylation site (G at position 2) which is required for membrane localization in fractionation experiments. This site is also required for the Arabidopsis avirulence and virulence functions of HopZ1a and HopZ2, respectively [15].

T3SEs typically show high levels of functional redundancy, making it difficult to dissect the role of individual effectors [21]. Transgenic expression of T3SEs has proven to be a powerful approach to overcome functional redundancy and probe the virulence functions of individual T3SEs [22]. To obtain a comprehensive picture of the virulence functions of the HopZ family in Arabidopsis, we created transgenic Arabidopsis inducible HopZ lines and analyzed these for HopZ-mediated immunomodulation. This work demonstrates that despite evolutionary diversification, all members of the HopZ family examined in this study can suppress the Arabidopsis PTI response.

## Materials and Methods

### Cloning

The HopZ1a, HopZ1b, HopZ1c, HopZ2 and HopZ3 wild type or C/A genes with an in-frame HA epitope tag were cloned into pBD as previously described [15]. The HopZ genes were cloned from the following *P. syringae* pathovars: HopZ1a from *syringae* A2, HopZ1b from *glycinea* UnB647, HopZ1c from *maculicola* ES4326, HopZ2 from *pisi* 895A, HopZ3 from *syringae* B728a [15]. For the YFP constructs, the HopZ or HopZ^C/A^ genes with an in-frame HA epitope tag were amplified by PCR to contain a 5′ *Xho*I site and a 3′ *Stu*I site. They were cloned into the pPIL vector to maintain the frame for the vector-encoded C-terminal HA-YFP fusion. pPIL was modified from pBD to contain an HA tag and a full-length YFP between the *Stu*I and *Spe*I sites. All constructs were confirmed by sequencing.

### Generation of Transgenic Plants

Col-0 plants were transformed with pBD::*hopZ*-HA or pBD::*hopZ^C/A^*-HA [15], [16] using the floral dip method. *zar1-1* plants were transformed with pBD::*hopZ1a*-HA or pBD::*hopZ1a^C/A^*-HA as previously described [15], [16], [19]. Transgenic plants were selected by Basta resistance in Sunshine #1 soil supplemented with 20∶20∶20 fertilizer. Transgenes were confirmed by PCR and sequencing. Homozygous T3 lines were identified by segregation ratios on plates containing half-strength Murashige and Skoog (MS) media and 6 mg/L bialophos. HopZ expression was tested by immunoblot analysis with an anti-HA antibody in the T1, T2 and T3 generations. Leaves were detached from the plants and floated on 30 µM dexamethasone or water for 48 or 96 hours, and frozen in liquid nitrogen. The leaf tissue was ground in a buffer containing 20 mM Tris pH 8.0, 100 mM NaCl, 1 mM DTT and 1% Triton X-100. The crude extract was cleared by centrifugation at 5000 g for 10 minutes at 4°C. SDS-PAGE loading dye was added to the samples and the samples were boiled for 5 minutes. 7.5 µL of protein was separated on 12% SDS-PAGE gels, blotted onto nitrocellulose membranes and detected using HA antibodies (Roche) by chemiluminescence (GE Healthcare). Photographs were taken 24 hours after spraying the plants with 30 µM dexamethasone or water.

Plants used in PTI and ETI suppression assays were also tested for HopZ expression. Plants were sprayed with 30 µM dexamethasone or water and harvested at 8 hours, 72 hours or 96 hours.

### *P. syringae* Infection Assays

The HopZ1a allele was amplified from the *P. syringae* pv. syringae A2, expressed under its native promoter and contained an in-frame HA tag at the C-terminus [15]. *P. syringae* pv. tomato DC3000 (*Pto*DC3000) carried pUCP20-P*_hopZ1a_*::*hopZ1a*-HA [15], pDSK519-P*_nptII_*:*AvrRpt2* [23], pVSP61-AvrPphB [24], pVSP61-AvrB [25], or pVSP61-AvrRpm1 [26].

HR and *in planta* growth assays were performed as has been described [15]. For infiltrations, *P. syringae* was resuspended to an OD_600_ = 0.1 (∼5×10^7^ cfu/mL) for HR assays, or diluted to 1×10^5^ cfu/mL for growth curves. Diluted inocula were hand-infiltrated using a needleless syringe as has been described [27]. Expression of the transgenic HopZ protein was induced 8 hours prior to infiltration. The HR was scored at 16–20 hours post-infiltration. For growth assays, 4 disks (1 cm^2^) were harvested, ground in 10 mM MgCl_2_, and plated on KB with rifampicin and cyclohexamide on day 0 and day 3 for colony counts. Expression of the transgenic HopZ protein was induced one hour after infiltration. Two-tailed homoschedastic t-tests were performed within genotypes to detect statistical significance.

### ROS Generation Assay

Measurement of ROS production was performed using a modified version of the luminol-based assay [2]. Plants were sprayed with 30 µM dexamethasone 24 hours before taking a series of 20 leaf disks (4 mm diameter) from the leaves of dexamethasone-treated or untreated 4 week-old HopZ transgenic plants. The disks were divided between two wells of a 96-well plate and washed with 200 µL of sterile water for 20 hours. The water was removed and replaced with 100 mM Tris HCl pH 8.0 containing 20 µg/ml horseradish peroxidase and 34 µg/ml luminol (44 hours after dexamethasone induction). Each matched pair of wells from a single plant was treated with either water or 2 µM flg22 peptide. Luminescence was measured for a 2 second interval every 2 minutes for a total of 50 minutes on an Infinite M1000Pro microplate reader (TECAN Group Ltd.) and the total summed. Each treatment was performed on three individual plants. The fold induction was calculated by dividing the mean value following flg22 induction by the mean value of the water control.

### MAPK Assays

Arabidopsis seedlings were grown for 11 days on a medium solidified with 0.8% agar that contained 0.5× MS salts with Gamborg's vitamins (M0404; Sigma) and then transferred to 6-well plates (six seedlings per well) in which each well contained 3 mL of liquid medium containing 0.5× MS salts with Gamborg's vitamins with 30 µM dexamethasone (for +Dex treatment) or 0.5× MS salt (−Dex treatment). Seedlings were gently shaken in 12 h light, 12 h dark cycle at day/night temperature regime of 22°C/18°C. 24 h after dexamethasone or mock application, 1 µM flg22 was added to each individual well after 2 h of light (subsequent to the dark cycle). Shaking was maintained for 20 minutes then seedlings were harvested and immediately frozen in liquid nitrogen. The 6 frozen seedlings were ground in liquid nitrogen and homogenized in 100 µL of extraction buffer (100 mM HEPES, pH 7.5, 5 mM EDTA, 5 mM EGTA, 2 mM dithiothreitol, 10 mM Na_3_VO_4_, 10 mM NaF, 50 mM ß-glycerolphosphate, 1 mM phenylmethylsulfonyl fluoride, and 10% glycerol, 1% (w/v) polyvinylpolypyrrolidone). After centrifugation at 13000 rpm for 30 min at 4°C, supernatants were frozen and stored at −20°C. The protein concentration was determined using a Bradford assay (BIO-RAD, Hercules, CA, USA). 20 µg of protein was separated in an 8% polyacrylamide gel. Immunoblot analysis was performed using anti-phospho-p44/p42 MAPK (1∶2000, Cell Signaling Technology, Danvers, MA) as primary antibody, and peroxidase-conjugated goat anti-rabbit IgG (1∶15000, A6154; Sigma).

## Results

### Generation of HopZ Transgenic Lines

**Figure 1:**
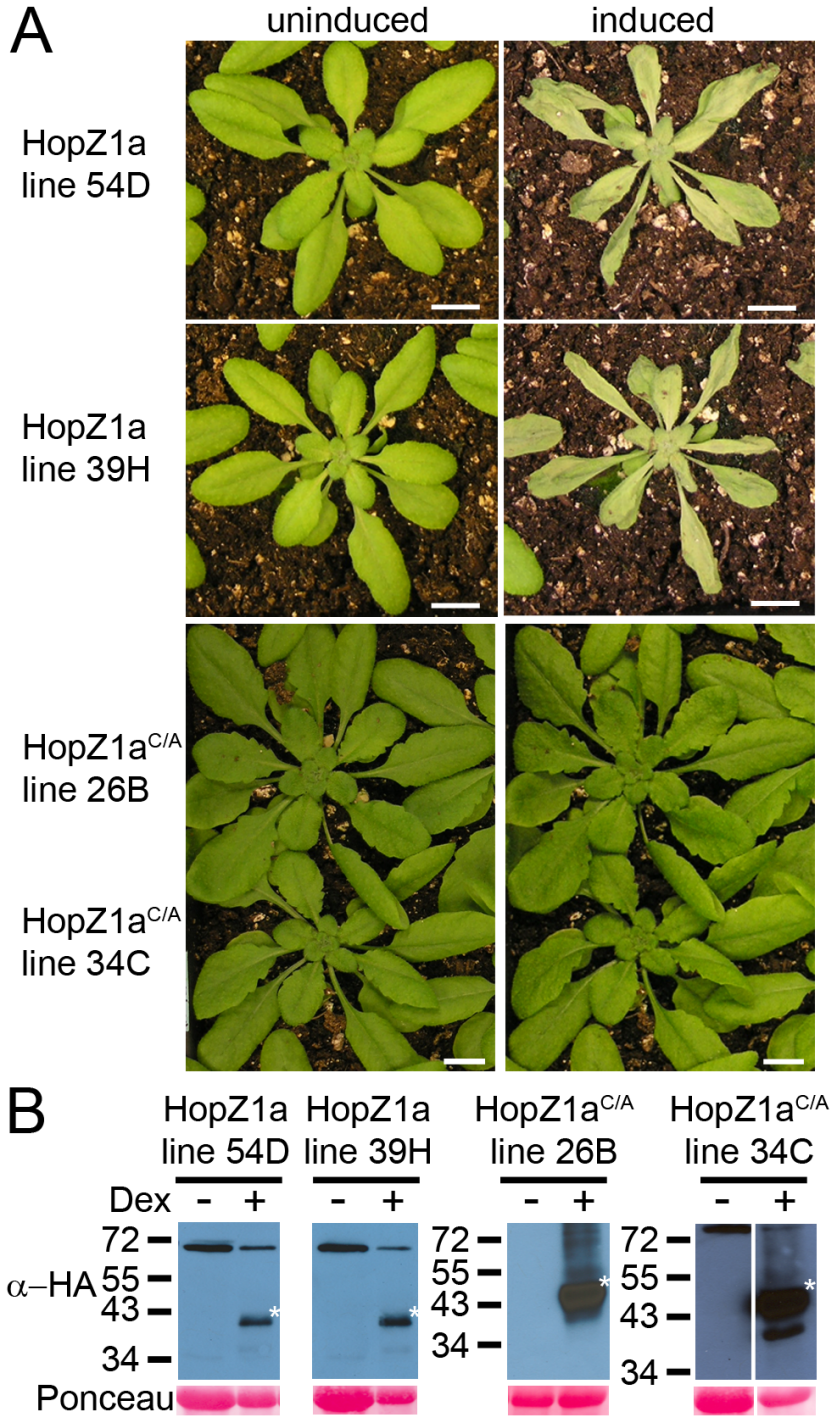
Transgenically expressed HopZ1a induces a whole plant HR that depends on the catalytic cysteine. (A) Phenotype of transgenic HopZ1a and HopZ1a^C/A^ plants (two independent transgenic lines shown for each genotype) 24 hours after transgene induction by spraying with 30 µM dexamethasone. The same individual for one line is shown before and after dexamethasone treatment. (B) Immunoblot analysis of HopZ1a and HopZ1a^C/A^ proteins expressed in Arabidopsis Col-0 transgenic lines after treatment with 30 µM dexamethasone or water. The Ponceau Red stained blot serves as the loading control. The expected size 42.1 kDa, and the expected band is marked with an asterisk.

We previously demonstrated that HopZ1a causes a strong HR in all infected Arabidopsis Col-0 leaves, while HopZ1b causes a weaker HR-like response in only 25% of leaves when delivered by *P. syringae* [15]. We also found that while HopZ1a-mediated ETI requires the ZAR1 NLR protein [16], the HR-like response induced by HopZ1b delivered by *P. syringae* is ZAR1-independent. To confirm these phenotypes, we generated transgenic Arabidopsis Col-0 plants expressing either HopZ1a or HopZ1b [16]. In agreement with previous findings, induction of the HopZ1a (Fig. 1A) or HopZ1b [16] transgenes by dexamethasone caused whole-plant HRs. We also confirmed our previous finding that the catalytic cysteine residue is required for recognition of both HopZ1a and HopZ1b *in planta* [15], [16], and mutation of this catalytic cysteine in transgenically expressed HopZ1a^C216A^ (hereafter HopZ1a^C/A^) (Fig. 1A, 1B) or HopZ1b^C212A^ (hereafter HopZ1b^C/A^) [16] resulted in a complete loss of the HR. This data corroborated phenotypes observed when the HopZs were delivered by *P. syringae* [15], [16], establishing the transgenic system as a viable alternative to study the effects of the HopZ proteins on plant immunity.

**Figure 2:**
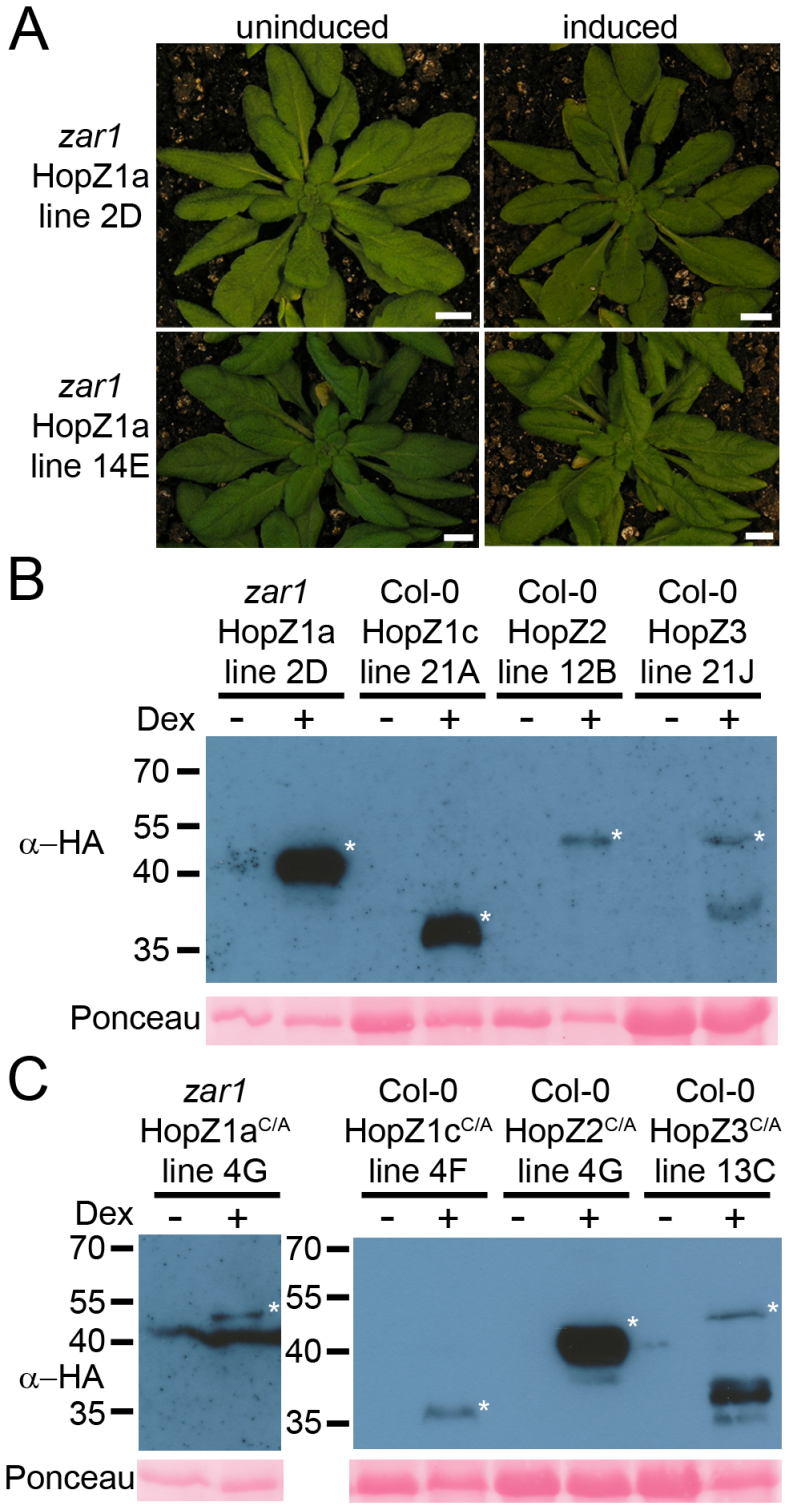
Transgenically expressed HopZ1a in Arabidopsis *zar1* background does not induce an HR. (A) Phenotype of transgenic HopZ1a and HopZ1a^C/A^ in *zar1-1* plants 24 hours after transgene induction by spraying with 30 µM dexamethasone. (B–C) Immunoblot analysis of HopZ (B) and HopZ^C/A^ (C) proteins expressed in transgenic lines after treatment with 30 µM dexamethasone or water. Transgenic HopZ1a is in a *zar1-1* background while HopZ1c, HopZ2 and HopZ3 are in a Col-0 background. The Ponceau Red stained blot serves as the loading control. The expected sizes are as follows: HopZ1a-HA 42.1 kDa, HopZ1c-HA 30.5 kDa, HopZ2-HA 41.9 kDa, HopZ3-HA 46.9 kDa, and the expected band is marked with an asterisk.

To further explore the virulence effects of HopZ1a in the absence of an ZAR1-mediated ETI response, we constructed transgenic HopZ1a-expressing *Arabidopsis* plants in the *zar1* background [16]. Since the HR-like response induced by HopZ1b delivered by *P. syringae* is ZAR1-independent we did not pursue this family member further in this study. We generated two independent transgenic lines for both wild-type HopZ1a and HopZ1a^C/A^ translationally fused C-terminally to a hemagglutinin (HA)-epitope tag. As expected, HopZ1a did not induce an HR when transgenically expressed in *zar1* plants (Fig. 2A). Protein expression of all lines was confirmed by western blotting (Fig. 2B, 2C; S1A, S1B Fig.).

In addition to HopZ1a, we generated two independent transgenic lines expressing wild type or catalytic mutants of HopZ1c, HopZ2, or HopZ3 in the Arabidopsis Col-0 background. We were only able to identify a single HopZ3^C/A^ line that continued to express in the T3 generation. We identified homozygous lines for each HopZ transgene, and confirmed dexamethasone-inducible protein expression for each line (Fig. 2B, 2C; S1A, S1B Fig.). Transgenic expression of each HopZ family member resulted in no macroscopic HR, consistent with our previous observations from *P. syringae*-delivered effectors into Arabidopsis (data not shown) [15].

### HopZ1a (in *zar1*), HopZ1c, HopZ2 and HopZ3 Actively Suppress PTI

**Figure 3:**
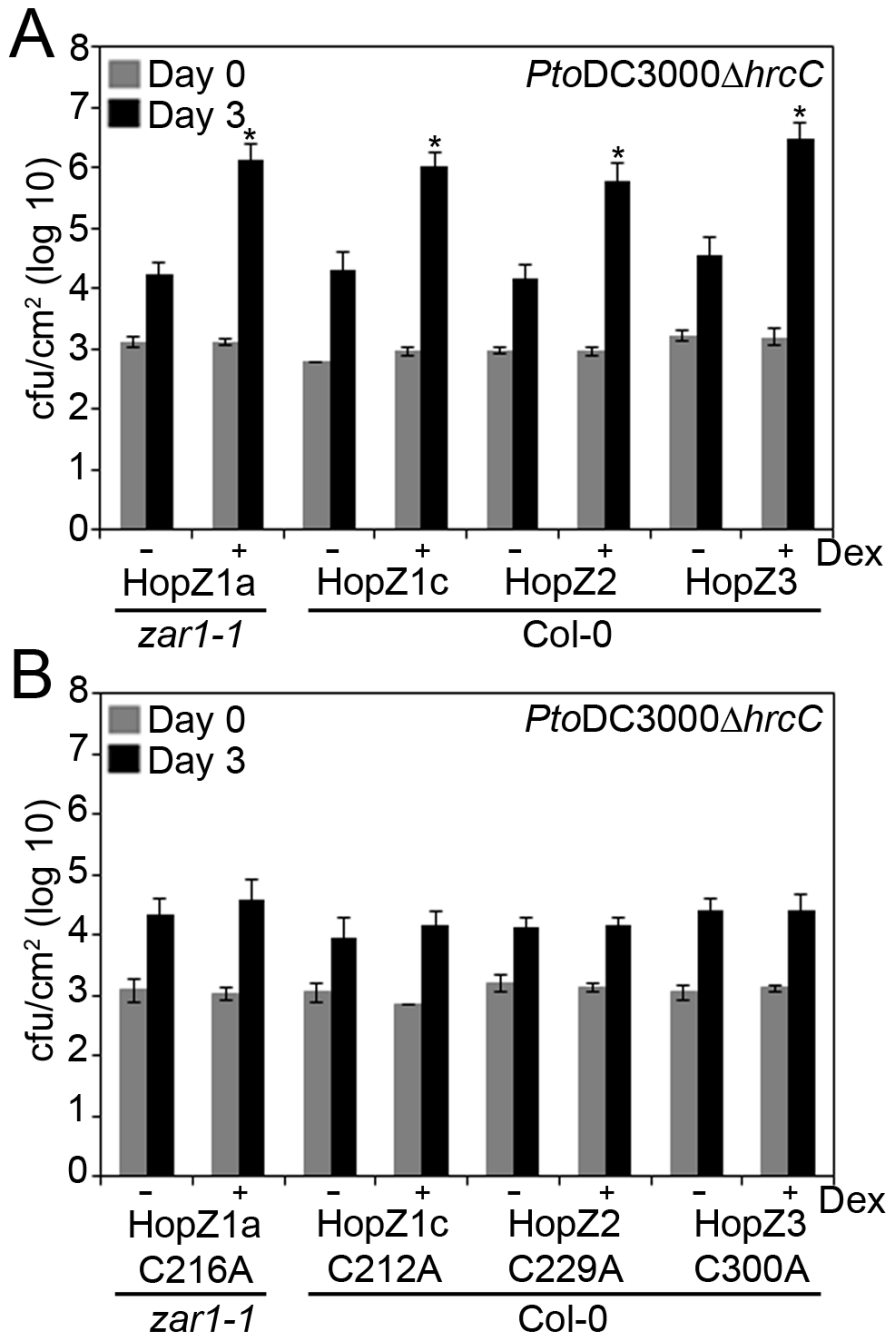
HopZ family members suppress PTI. (A) *Pto*DC3000Δ*hrcC* was pressure infiltrated at 10^5^ cfu/mL into transgenic HopZ1a (line 2D), HopZ1c (line 21A), HopZ2 (line 12B) or HopZ3 (line 21J). The HopZ1a transgenic line was generated in an Arabidopsis *zar1-1* background to focus on virulence rather than avirulence response, while the other members of the HopZ family were generated in *Arabidopsis* Col-0. Bacterial counts were determined one hour post-infection (Day 0) and 3 days post-infection (Day 3). Transgenic HopZ lines were induced by spraying with 30 µM dexamethasone or water 1 hour post-infiltration. Two-tailed homoschedastic t-tests were performed to test for significant differences. Within a plant genotype, dexamethasone-induced plants were compared to non-induced plants and significant differences are indicated by an asterisk (* P<0.01). Error bars indicate the standard deviation from the mean of 10 samples. Growth assays were performed at least 3 times. (B) Transgenic HopZ1a^C/A^ (line 1G), HopZ1c^C/A^ (line 4F) and HopZ3^C/A^ (line 13C) lines were tested as in part A.,Transgenic HopZ2^C/A^ (line 4G) line was sprayed 24 hours pre-infiltration as its expression level was lower than the other lines.

We tested the ability of transgenic HopZ1a, HopZ1c, HopZ2 and HopZ3 T3SEs to suppress PTI by infiltrating transgenic plants with the *P. syringae* pv. tomato DC3000Δ*hrcC* mutant (hereafter *Pto*DC3000Δ*hrcC*), that lacks a critical structural component of the T3SS [28]. Since *Pto*DC3000Δ*hrcC* cannot deliver T3SEs to the plant and suppress PTI, it grows very poorly in Arabidopsis. If the transgenic HopZ T3SE can suppress PTI, higher *Pto*DC3000Δ*hrcC* growth should be observed. Expression of transgenic HopZ was induced 1 hour after infiltrating the plants with *Pto*DC3000Δ*hrcC*, and bacterial growth was measured after three days. All dexamethasone-induced HopZ lines showed a ∼2 log increase in *Pto*DC3000Δ*hrcC* growth compared to non-induced HopZ lines in two independent transgenic lines (Fig. 3A, S2A Fig.), indicating that each of the four HopZ effector proteins is able to suppress PTI.

To determine if the observed PTI suppression is catalytic dependent, we tested transgenic HopZ^C/A^ lines for their ability to support *Pto*DC3000Δ*hrcC* growth. Dexamethasone-induced plants for each of the HopZ1a^C/A^, HopZ1c^C/A^, HopZ2^C/A^ and HopZ3^C/A^ lines exhibited the same level of *Pto*DC3000Δ*hrcC* growth as was observed in the non-induced HopZ^C/A^ lines, in two independent transgenic lines (Fig. 3B; S2B Fig.). As some HopZ^C/A^ lines exhibited lower levels of protein production than the corresponding HopZ wild type line, we cannot exclude the possibilility that this is due to the level of protein produced. However we also observed strongly reproducible phenotypes in two independent lines for the same T3SE. Therefore, we conclude that the ability of HopZ T3SEs to suppress PTI requires the HopZ enzymatic function.

### Hopz1a, Hopz2 and Hopz3 Actively Block Production of Reactive Oxygen Species

**Figure 4:**
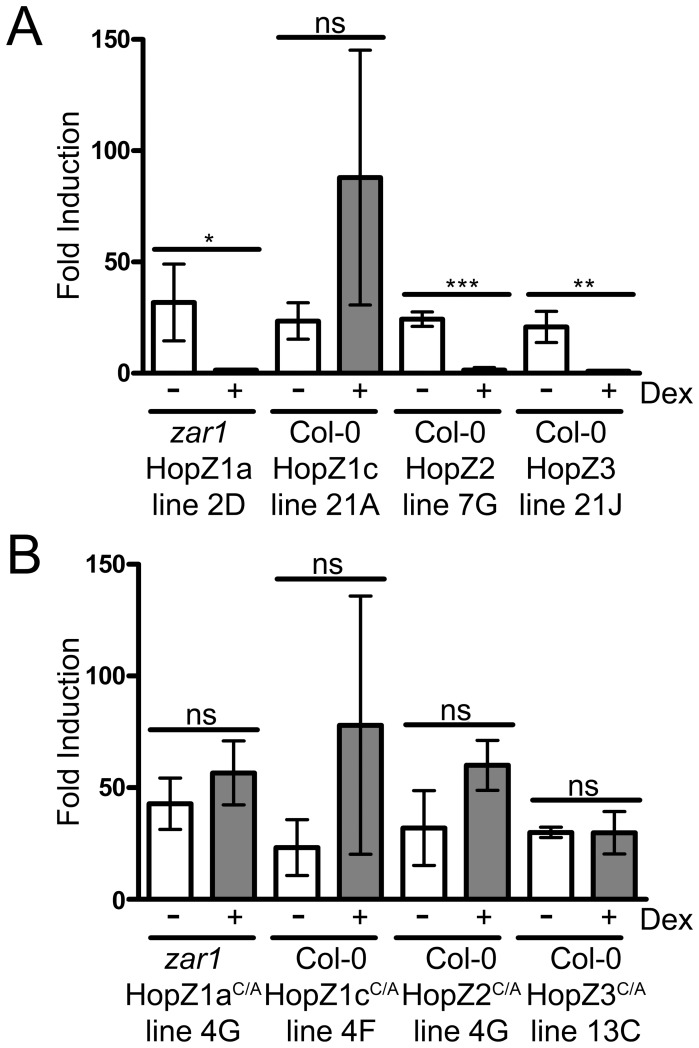
HopZ family members differentially suppress ROS production. Transgenic HopZ 4 week-old plants were induced with 30 µM dexamethasone or mock treated with water 24 hours before sampling tissue. Tissue was treated with 2 µM flg22 44 hours after dexamethasone induction. ROS production was measured using a luminol-dependent chemiluminescence assay. Luminescence was measured for a total of 100 seconds over a 50 minute period from 3 plants per treatment. Each flg22-treated sample was normalized with the paired water treated sample to give a fold induction. Two-tailed homoschedastic t-tests were performed to test for significant differences. Within a plant genotype, dexamethasone induced plants were compared to non-induced plants and significant differences are indicated by an asterisk (* P<0.05, ** P<0.01, *** P<0.001, ns = not significant). Error bars indicate the standard deviation from the mean. Similar results in observed in two experiments. (A) Transgenic HopZ lines in *zar1* (HopZ1a) or Col-0 (HopZ1c, HopZ2, HopZ3) backgrounds. (B) Transgenic catalytically inactive HopZ^C/A^ lines in *zar1* (HopZ1a^C/A^) or Col-0 (HopZ1c^C/A^, HopZ2^C/A^, HopZ3^C/A^) backgrounds.

To determine if the HopZ family members can suppress the production of reactive oxygen species, we performed a plate-based assay using flg22 to induce PTI in leaf discs. ROS production was measured by luminol-dependent chemiluminescence. Transgenic HopZ1a, HopZ2 and HopZ3 plants were induced with dexamethasone 24 hours before harvesting the tissue for the ROS assay. In non-dexamethasone treated plants, application of flg22 strongly induced ROS production, as observed in previous reports [2]. However in dexamethasone-induced plants, HopZ1a, HopZ2 and HopZ3 strongly suppressed the production of ROS, while HopZ1c was unable to suppress ROS production (Fig. 4A, see discussion). We also tested HopZ^C/A^ transgenic lines to determine whether HopZ catalytic activity was necessary for the suppression of ROS production. flg22 application caused ROS production in HopZ1a^C/A^, HopZ1c^C/A^, HopZ2^C/A^, and HopZ3^C/A^ lines (Fig. 4B). Col-0 plants did not exhibit significant differences in ROS production after flg22 induction between non-treated or dexamethasone-treated plants (S3 Fig.). Therefore, HopZ1a, HopZ2 and HopZ3 each require the catalytic cysteine to block ROS production.

### HopZ1a, HopZ2 and HopZ3 Suppress MAP Kinase Activation

**Figure 5:**
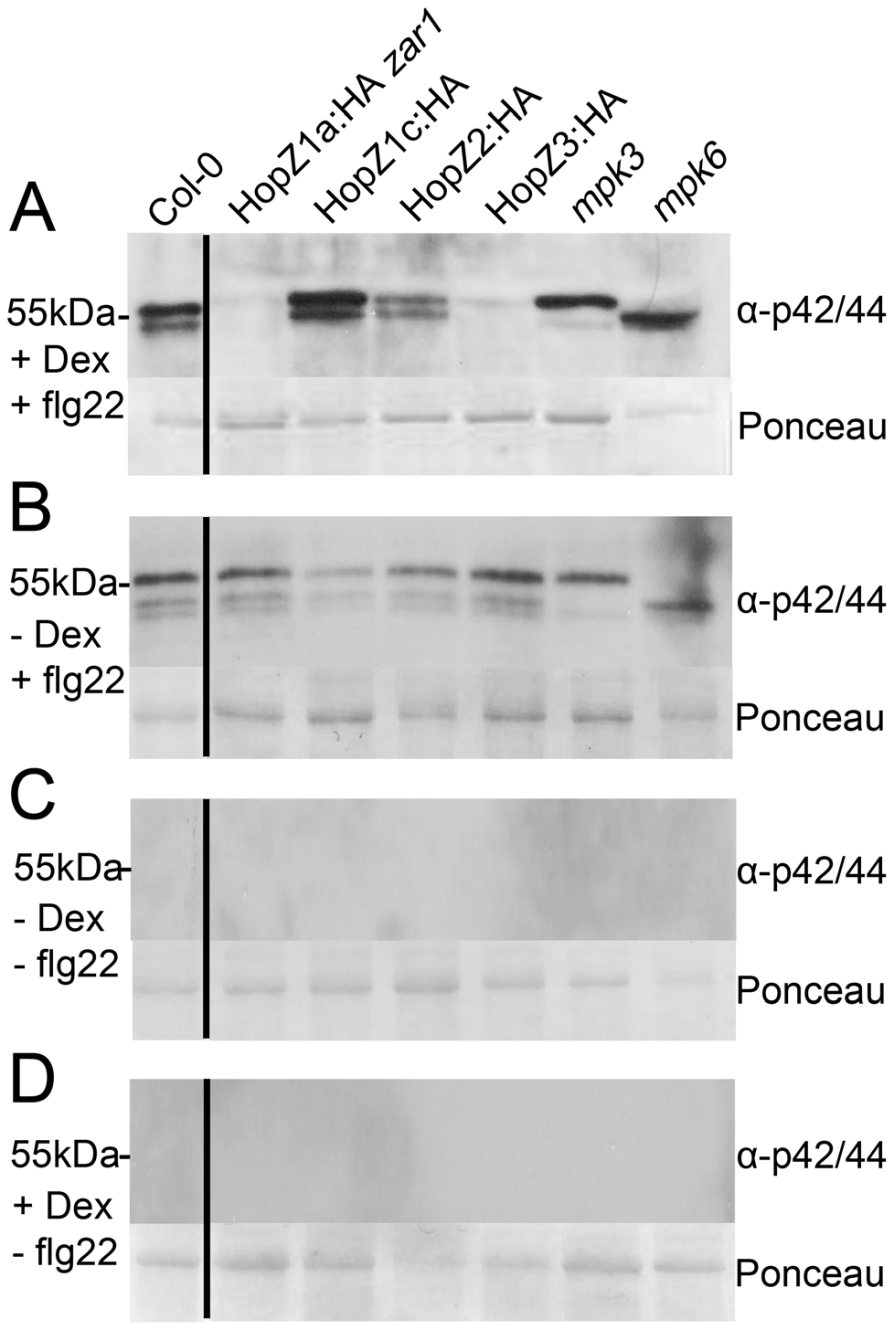
HopZ family members differentially suppress MPK phosphorylation. Transgenic HopZ 11 day-old seedlings were induced with 30 µM dexamethasone or mock treated with water 24 hours prior to application of 1 µM flg22 and harvested 20 mins after flg22 application. 20 µg of seedling tissue was subjected to immunoblot analysis with phospho-specific p42/p44 MPK antibodies. *mpk3* or *mpk6* are mutants in one of the two MPKs that are phosphorylated. The Ponceau Red stained blot serves as the loading control. The following lines were tested: HopZ1a line 2D in *zar1*, HopZ1c line 32A in Col-0, HopZ2 line 12B in Col-0, and HopZ3 line 21J in Col-0. The experiment was repeated three times with similar results. (A) +Dex+flg22 (B) −Dex+flg22 (C) −Dex−flg22 (D) +Dex−flg22.

MAMP recognition by PRRs leads to a MAPK signaling cascade, and often the phosphorylation of MPK3 and MPK6 [29]. Several T3SEs interfere with kinase cascades to block immunity [6]. To determine whether the HopZ family members can block phosphorylation of MAPK cascades, we treated seedlings with dexamethasone or water, induced PTI with the flg22 peptide 24 hours post-dexamethasone treatment, and harvested the tissue for immunoblots. We used the phospho-specific p42/p44 antibody to determine if phosphorylation of MPK3 and MPK6 were suppressed by any of the HopZ family members. Interestingly, we found that transgenically expressed HopZ1a and HopZ3 strongly suppressed phosphorylation of both MPK3 and MPK6, while HopZ2 only weakly suppressed their phosphorylation (Fig. 5A). HopZ1c did not affect the phosphorylation status of MPK3 and MPK6. As expected, flg22-treated Col-0 plants showed strong phosphorylation of MPK3 and MPK6. In uninduced HopZ transgenic lines, flg22 treatment induced phosphorylation of MPK3 and MPK6 (Fig. 5B). MPK3/6 phosphorylation was not observed in flg22-untreated samples (Fig. 5C, 5D). Therefore, we conclude that HopZ1a, HopZ2 and HopZ3 suppress MAPK phosphorylation.

### The HopZ Family Cannot Suppress AvrB, AvrPphB, AvrRpm1 and AvrRpt2 ETI in Arabidopsis

In addition to PTI-suppression, transgenic expression of T3SEs has also been demonstrated to block ETI [30], [31]. To investigate whether any of the HopZ family members could suppress ETI induced by other T3SEs, we induced HopZ expression 8 hours prior to infiltrating the plants with *Pto*DC3000 carrying AvrB, AvrRpm1, AvrRpt2, AvrPphB or HopZ1a. Since ZAR1 is required for the HopZ1a-induced HR, we did not see a HopZ1a HR in HopZ1a-expressing *zar1* plants (Table 1). AvrB, AvrPphB, AvrRpm1 and AvrRpt2 still induced an HR in untreated Arabidopsis Col-0 or *zar1-1* backgrounds (Table 1), consistent with previous observations [16]. We observed similar numbers of leaves with an HR in response to each of the T3SEs, in dexamethasone-treated or untreated plants (Table 1). In all HopZ transgenic lines, HopZ protein was detectable by immunoblot analysis by 8 hours post-dexamethasone induction (S4 Fig.). This indicates that the HopZ family members cannot suppress the HR from the unrelated T3SEs AvrB, AvrRpm1, AvrRpt2 or AvrPphB, nor the related T3SE HopZ1a.

**Table 1:** Number of leaves showing an HR when infiltrated with *Pto*DC3000 carrying different recognized T3SEs in transgenic HopZ *Arabidopsis* lines.

Transgenic line	Dex induction	MgCl_2_	EV	HopZ1a	AvrB	AvrRpm1	AvrPphB	AvrRpt2
*zar1-1 hopz1a* 2D	−	0/10	0/10	0/10	7/10	7/10	10/10	10/10
*zar1-1 hopz1a* 2D	+	0/10	0/10	0/10	8/10	9/10	8/10	10/10
*zar1-1 hopz1a* 14E	−	0/9	0/9	0/7	8/9	9/9	9/9	9/9
*zar1-1 hopz1a* 14E	+	0/9	0/9	0/8	9/9	9/9	9/9	9/9
*hopz1c* 21A	−	0/10	0/10	7/10	9/9	10/10	10/10	10/10
*hopz1c* 21A	+	0/6	0/6	6/6	6/6	6/6	6/6	6/6
*hopz1c* 32A	−	0/10	0/10	10/10	10/10	10/10	10/10	10/10
*hopz1c* 32A	+	0/10	0/10	10/10	10/10	10/10	10/10	10/10
*hopz2* 12B	−	0/10	0/10	10/10	10/10	10/10	10/10	10/10
*hopz2* 12B	+	0/10	0/10	10/10	10/10	9/10	9/10	10/10
*hopz2* 7G	−	0/10	0/10	10/10	10/10	10/10	10/10	10/10
*hopz2* 7G	+	0/10	0/10	10/10	10/10	10/10	9/10	10/10
*hopz3* 21J	−	0/10	0/10	10/10	10/10	10/10	10/10	10/10
*hopz3* 21J	+	0/9	0/9	9/9	8/8	9/9	8/8	9/9
*hopz3* 36A	−	0/10	0/10	10/10	10/10	10/10	10/10	10/10
*hopz3* 36A	+	0/10	0/10	9/9	10/10	9/9	10/10	10/10

### HopZ1a, HopZ1b, HopZ1c and HopZ2 Localize to the Vicinity of the Plasma Membrane While HopZ3 Is Primarily Nuclear Localized

**Figure 6:**
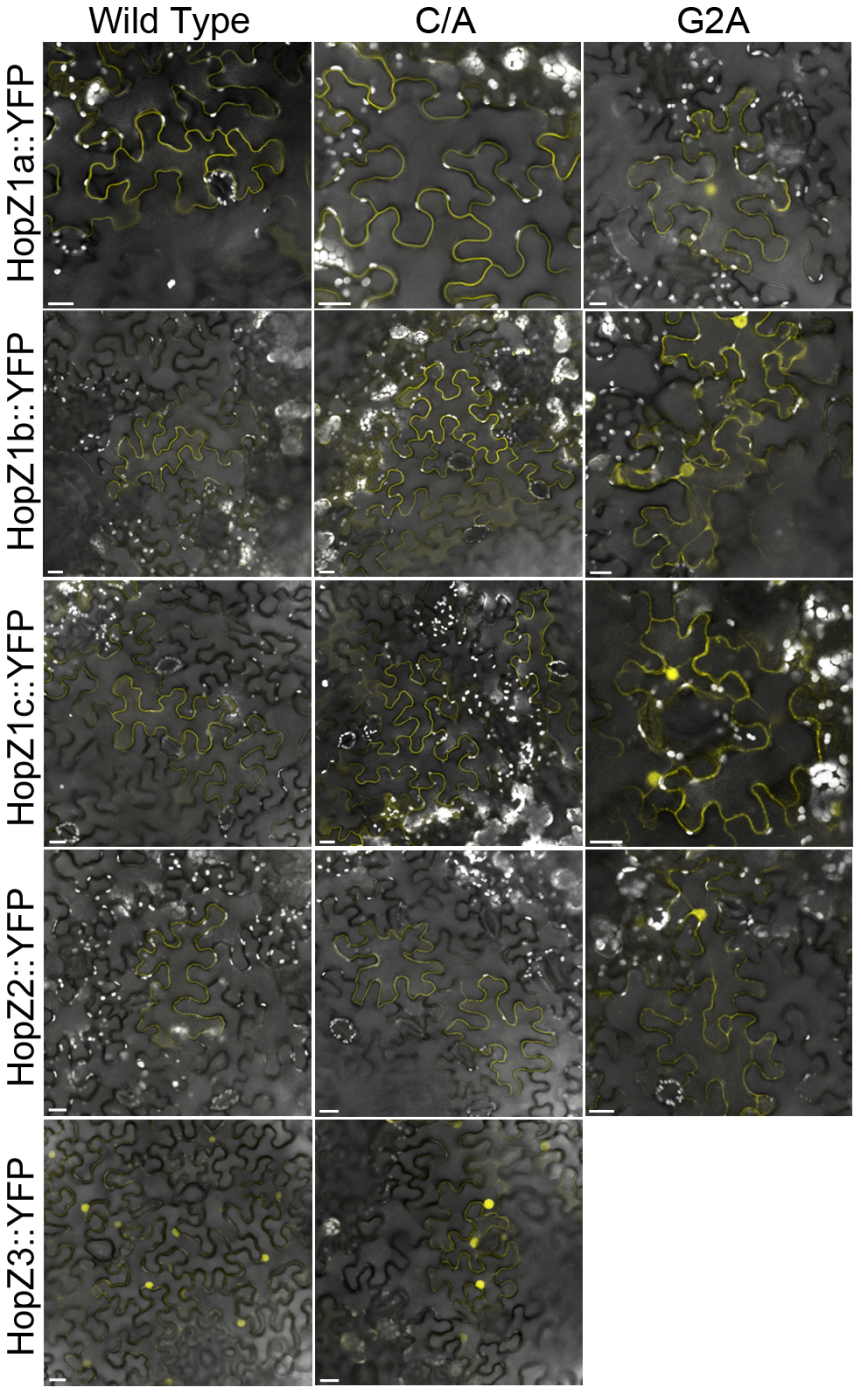
HopZ1a, HopZ1b, HopZ1c and HopZ2 localize to the vicinity of the plasma membrane while HopZ3 is primarily nuclear localized. *Agrobacterium tumefaciens* carrying HopZ-YFP constructs was pressure-infiltrated into *Nicotiana benthamiana* leaves, and expression was induced by spraying leaves with 30 µM dexamethasone 24 hours post-infiltration. Leaves were imaged 24–48 hours after dexamethasone induction. C/A indicates the catalytic cysteine was mutated to an alanine. G2A indicates the myristoylated glycine was mutated to an alanine.

We showed previously that HopZ1a, HopZ1b, HopZ1c and HopZ2 fractionate with membranes and require an N-terminal glycine for membrane association, while HopZ3 is soluble and lacks the N-terminal glycine [15]. To more specifically determine the subcellular localization of each of the HopZ alleles, we conducted confocal microscopy on transiently expressed YFP-tagged HopZ proteins in *Nicotiana benthamiana*. HopZ1a, HopZ1b, HopZ1c and HopZ2 all localized to the cell periphery in the vicinity of the plasma membrane (Fig. 6). Mutation of the catalytic cysteine in each of these did not affect localization of the protein (Fig. 6). HopZ3 and HopZ3^C/A^ both localized primarily to the nucleus. The diffuse fluorescence observed in the cell periphery is more likely to be cytoplasmic because we previously showed that HopZ3 was a soluble protein [15]. Mutation of the N-terminal glycine in the HopZ1 alleles and HopZ2 resulted in more diffuse fluorescence throughout the cell, nucleus and a structure reminiscent of the endoplasmic reticulum, in addition to the cell periphery (Fig. 6).

## Discussion

We used a transgenic approach to systemically examine modulation of the Arabidopsis immune system by the evolutionarily diverse HopZ family. All of the HopZ family members tested were able to promote virulence of a T3SS-deficient strain (Fig. 3). This is remarkable since only HopZ1a and HopZ2 have been demonstrated to promote apoplastic *P. syringae* growth in Arabidopsis when delivered from the bacteria [15], [16]. The novel virulence functions observed for HopZ1c and HopZ3 attest to the power of transgenic expression of T3SEs to uncover immunomodulatory functions for T3SEs. For all of the HopZ members, *P. syringae* growth promotion was lost in Arabidopsis transgenic lines expressing the corresponding catalytic mutant, indicating that the catalytic activity of the HopZ member was necessary for its virulence function. Interestingly, HopZ members appear to differentially suppress the Arabidopsis immune response since HopZ1a, HopZ2 and HopZ3 all blocked PTI-induced ROS and MAP kinase activation whereas HopZ1c did not.

Diversification of PTI-suppression mechanisms by the HopZ family is also supported by differences in subcellular localization. HopZ1a, HopZ1b, HopZ1c and HopZ2 localize to the cell periphery (likely the plasma membrane), whereas HopZ3 localizes primarily to the nucleus (Fig. 6) [15], [20], [32]. This would suggest that the virulence targets of HopZ1 and HopZ2 are primarily found at the plasma membrane, while the virulence targets of HopZ3 are primarily nuclear. We did not observe nuclear localization of HopZ1a as reported in [20], [33]. Barring differences in plant growth conditions, this may be due to the differences between the 35S promoter driven system used in these studies and our dexamethasone-inducible vector. Note that we have observed nuclear-like localization of HopZ1a when interacting with the NLR resistance protein ZAR1 which supports nuclear localization of HopZ1a under certain conditions [17]. We observed significant differences in localization between HopZ3 and other HopZ family members that were dependent on the predicted myristoylation site (Fig. 6) [15]. Despite subcellular localization differences, HopZ1a, HopZ2 and HopZ3 all suppressed ROS production as well as MAP kinase activation induced by the PTI elicitor flg22. The different localization patterns of HopZ1a and HopZ2 versus HopZ3 suggest that they may suppress these PTI-hallmarks via different cellular targets.

Numerous targets have been identified for HopZ family members in Arabidopsis [13]. HopZ1a acetylates tubulin and destabilizes microtubule networks in planta [19], and acetylates the ZED1 pseudokinase, resulting in the initiation of ZAR1-mediated immunity [17], [34]. Since the deletion of ZED1 suppressed the avirulence function of HopZ1a but did not affect its virulence function, we proposed that ZED1 acts as a decoy to trap HopZ1a into the ZAR1 immune complex [17]. ZED1 is a member of an evolutionarily conserved family of ZED1-related kinases (ZRKs) and pseudokinases, some of which are co-localized along the Arabidopsis chromosome. Preliminary data indicates that HopZ1a can also acetylate a number of ZRK family members (Guttman and Desveaux, unpublished). HopZ1a can also acetylate and promote the degradation of the JAZ transcriptional repressors involved in jasmonic acid signaling [33]. HopZ2 directly interacts with MLO2, a barley susceptibility factor, which presumably stabilizes MLO2 and promotes bacterial virulence [35]. The recently described HopZ4 family member targets the proteasomal subunit RPT6 and disrupts proteasomal function [14]. In soybean, HopZ1a and HopZ1b interact with and inhibit a 2-hydroxyisoflavanone dehydratase (GmHID1) that contributes to plant defense [20]. These results demonstrate the diversity of proteins that can be targeted by the HopZ family. It remains to be determined whether any of these represent operative targets by which the HopZ members can suppress the PTI response at the level of MAP kinase signaling and/or ROS production.

The large number of targets found for HopZ1a raises the possibility that it may acetylate a diverse range of kinases to block immunity and/or disrupt the homeostasis of the host cell. This potential for target promiscuity (as opposed to specificity) would intuitively appear to be a robust strategy for a pathogen to take; however these hypothetical kinase targets have not yet been identified. One critically important family of kinases commonly targeted by pathogens is the immune MAP kinases [10]. In fact, the archetypal member of the YopJ/HopZ superfamily, YopJ, is an acetyltransferase that blocks phosphorylation of MAPK kinases in the binding pocket, resulting in the inhibition of innate immunity [13], [34], [36], [37], [38]. It will be particularly interesting to determine if any HopZ T3SEs can specifically acetylate the plant immune MAPKs (MPK3 and MPK6) as a mechanism to disrupt their signaling.

The ability of HopZ1c to suppress PTI is intriguing and represents the first *in vivo* function ascribed to this T3SE. HopZ1c is found in *P. syringae* pv. maculicola ES4326 and represents the only HopZ member isolated from a *P. syringae* pathovar with demonstrated virulence in Arabidopsis. HopZ1c is 97% identical to HopZ1b up to a C-terminal frameshift mutation that results in 19 amino acids followed by a premature stop codon that truncates the protein by 30% relative to HopZ1b. Despite this truncation HopZ1c retains the catalytic triad and the catalytic cysteine was required for its ability to block PTI (Fig. 3). This raises the possibility that the C-terminal third of the HopZ family is dispensable for acetyltransferase activity or that HopZ1c displays another function that requires the catalytic triad. A crystal structure of the YopJ/HopZ superfamily is still lacking, but the C-terminus of YopJ has been proposed to potentially harbor the acetyl coenzyme A binding site [39]. If this is the case for the HopZ family, then HopZ1c must display additional enzyme activity. It is noteworthy that HopZ1c was shown to possess weak *in vitro* protease activity against a generic substrate (casein) that was dependent on the catalytic cysteine [12]. HopZ1c may thus use water instead of acetyl coenzyme A during its enzymatic reaction, resulting in hydrolysis of substrates rather than acetylation [39]. Uncovering this enzymatic activity will await the identification of HopZ1c targets. These likely include PTI-signaling components downstream of ROS production and MAP kinase activation since these events are still intact in HopZ1c expressing plants (Figs. 4, 5).

None of the HopZ family members were able to suppress ETI-associated HR from related or unrelated T3SEs (Table 1). Previous work showed that HopZ1a and AvrRpt2 have partially additive effects in restricting bacterial growth, using a competitive index assay, suggesting that HopZ1a and AvrRpt2 may share common immune signaling components or interfere with the ETI of one another [40]. Our results would support the former explanation since HopZ1a expression did not interfere with the AvrRpt2-induced HR. However, these common components would be novel since we have demonstrated that ZAR1-mediated ETI does not require known components of RPS2 signaling: salicylic acid, NDR1 and RAR1 [16]. It is also possible that AvrRpt2 interferes with HopZ1a induced ETI, which could be revealed by transgenic expression of AvrRpt2 followed by HopZ1a-ETI induction. Furthermore, although no differences in ETI-associated macroscopic HR could be detected in HopZ-expressing plants, it is possible that differences might be detected using quantitative assays such as conductivity measurements or bacterial growth assays.

Zhou and colleagues demonstrated that HopZ1b can suppress the HopZ3-induced HR in *N. benthamiana* [41]. Although we could not test HopZ1b, we found that transgenically expressed HopZ1c, HopZ2 and HopZ3 did not interfere with HopZ1a ETI (Table 1). This may reflect a HopZ1b-specific ETI-suppression response or may be due to differences in the ETI responses induced by HopZ3 versus HopZ1a. HopZ3 can suppress the HR triggered by AvrPto1, HopAA1, HopM1 and HopAE1 when transiently expressed in *N. benthamiana* [18]. In Arabidopsis, HopZ3 was unable to interfere with ETI of AvrB, AvrRpm1, AvrPphB, AvrRpt2 or HopZ1a (Table 1), suggesting important differences between 1) these sets of ETI responses, and/or 2) the *N. benthamiana* and Arabidopsis systems.

Overall, our work reinforces that transgenic T3SE lines are powerful tools to probe the plant immune system, and to dissect the virulence functions of individual T3SEs. This work further highlights how an evolutionarily diverse T3SE can suppress the Arabidopsis immune response using diverse mechanisms. This is striking since only HopZ1c is native to a *P. syringae* pathovar that is virulent on Arabidopsis. HopZ1a, HopZ2 and HopZ3 were isolated from pathogens of ornamental pear, pea and beans, respectively. This emphasizes that T3SEs from diverse origins can be used to probe the immune systems of any plant species, not necessarily only the host of the original pathogen.

T3SE functions observed in transgenic plants may or may not represent their function when delivered from the bacteria. Nevertheless, these functions represent what T3SEs “can” do and what their functions “could” potentially be. Analogous to synthetic biology, which aims “to extend the study of biological systems beyond those that exist”, the study of T3SEs should endeavor to fully explore the functions of these powerful molecules in natural and artificial systems in order to effectively explore their potential as probes to manipulate and engineer biological systems [42].

## Supporting Information

S1 Fig **HopZ and HopZ^C/A^ proteins are expressed in transgenic Arabidopsis lines.** Immunoblot analysis of HopZ (A) and HopZ^C/A^ (B) proteins expressed in transgenic lines after treatment with 30 µM dexamethasone or water. Transgenic HopZ1a is in a *zar1-1* background while HopZ1c, HopZ2 and HopZ3 are in a Col-0 background. The Ponceau Red stained blot serves as the loading control. The expected sizes are as follows: HopZ1a-HA 42.1 kDa, HopZ1c-HA 30.5 kDa, HopZ2-HA 41.9 kDa, HopZ3-HA 46.9 kDa, and the expected band is marked with an asterisk.
(TIF)

S2 Fig **HopZ family members suppress PTI.** (A) *Pto*DC3000Δ*hrcC* was pressure infiltrated at 1×10^5^ cfu/mL into transgenic HopZ1a in *zar1-1* (line 14E), or HopZ1c (line 32A), HopZ2 (line 7G) or HopZ3 (line 36A) in *Arabidopsis* Col-0. Bacterial counts were determined one hour post-infection (Day 0) and 3 days post-infection (Day 3). Transgenic HopZ lines were sprayed with 30 µM dexamethasone or water 1 hour post-infiltration. Two-tailed homoschedastic t-tests were performed to test for significant differences. Within a plant genotype, dexamethasone-induced plants were compared to non-induced plants and significant differences are indicated by an asterisk (* P<0.01). Error bars indicate the standard deviation from the mean of 10 samples. Growth assays were performed at least 3 times. (B) Transgenic HopZ1a^C/A^ (line 4G), and HopZ1c^C/A^ (line 40F) lines were tested as in part A. Transgenic HopZ2^C/A^ (line 5C) was sprayed 24 hours pre-infiltration as its expression level was lower than the other lines. We were unable to identify a second HopZ3^C/A^ line that continued to express in the T3 generation.
(TIF)

S3 Fig **Dexamethasone does not affect ROS production in Arabidopsis Col-0 after flg22 induction.** Untransformed Col-0 4 week-old plants were induced with 30 µM dexamethasone or mock treated with water 24 hours before sampling tissue. Tissue was treated with 2 µM flg22 44 hours after dexamethasone induction. ROS production was measured using a luminol-dependent chemiluminescence assay. Luminescence was measured for a total of 100 seconds over a 50 minute period from 3 plants per treatment. Each flg22-treated sample was normalized with the paired water treated sample to give a fold induction. Two-tailed homoschedastic t-tests were performed to test for significant differences. Within a plant genotype, dexamethasone-induced plants were compared to non-induced plants and no significant differences were observed (ns = not significant). Error bars indicate the standard deviation from the mean. Similar results were observed in two experiments.
(TIF)

S4 Fig **Transgenic HopZ family members cannot suppress ETI from related or unrelated T3SEs.** Immunoblot analysis of HopZ proteins expressed in transgenic lines 8 hours after treatment with 30 µM dexamethasone or water. Transgenic HopZ1a is in a *zar1-1* background while HopZ1c, HopZ2 and HopZ3 are in a Col-0 background. The Ponceau Red stained blot serves as the loading control. The expected sizes are as follows: HopZ1a-HA 42.1 kDa, HopZ1c-HA 30.5 kDa, HopZ2-HA 41.9 kDa, HopZ3-HA 46.9 kDa, and the expected band is marked with an asterisk.
(TIF)

S1 Table **GenBank accession numbers and** ***P. syringae*** **strain for each member of the HopZ family.**
(DOC)
