# Supplementary figures and images for: Immunomodulation by the Pseudomonas syringae HopZ Type III Effector Family in Arabidopsis
Source: PLoS One. 2014 Dec 29;9(12):e116152. doi: 10.1371/journal.pone.0116152 (PMC4278861; doi:10.1371/journal.pone.0116152)

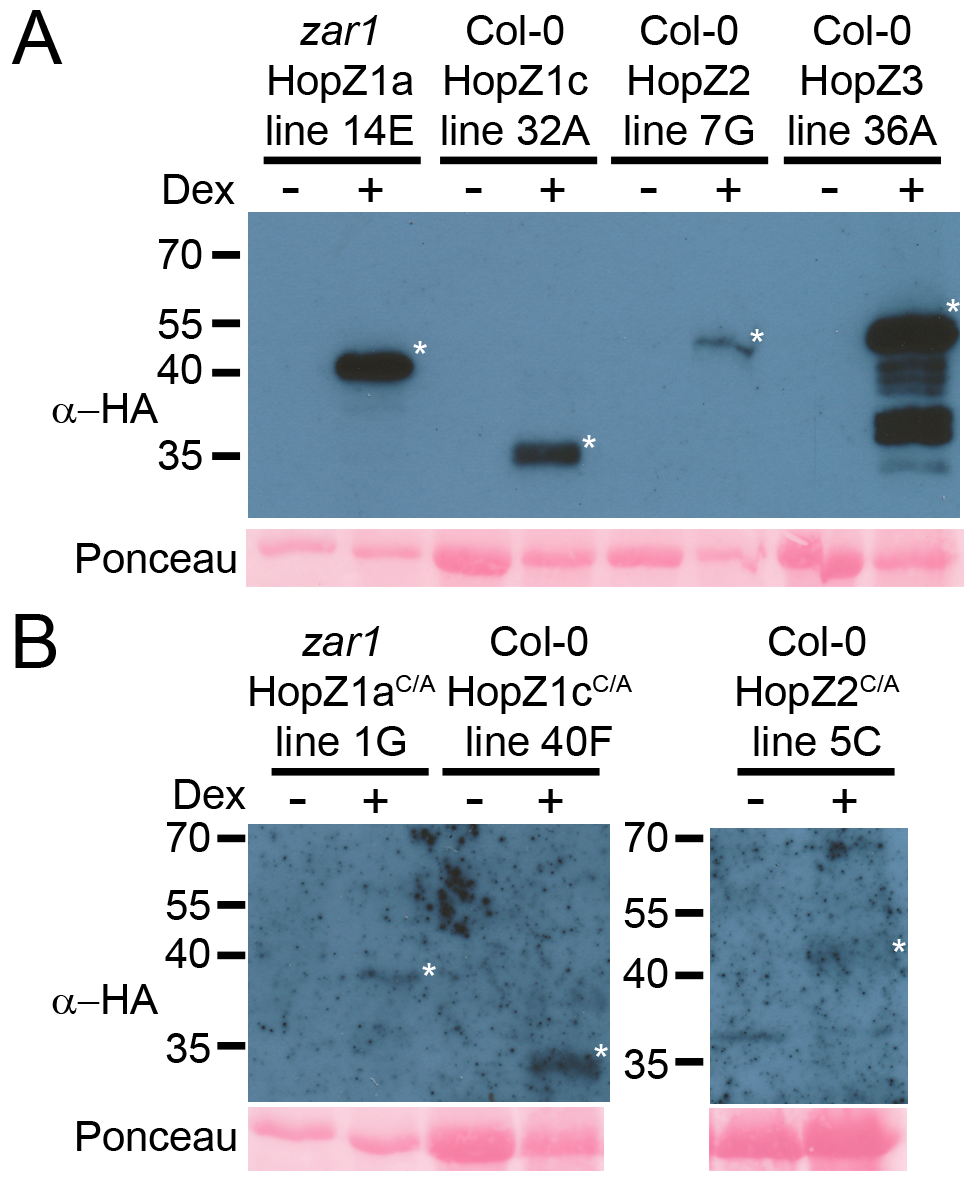

Supplement: S1 Fig — HopZ and HopZC/A proteins are expressed in transgenic Arabidopsis lines. Immunoblot analysis of HopZ (A) and HopZC/A (B) proteins expressed in transgenic lines after treatment with 30 µM dexamethasone or water. Transgenic HopZ1a is in a zar1-1 background while HopZ1c, HopZ2 and HopZ3 are in a Col-0 background. The Ponceau Red stained blot serves as the loading control. The expected sizes are as follows: HopZ1a-HA 42.1 kDa, HopZ1c-HA 30.5 kDa, HopZ2-HA 41.9 kDa, HopZ3-HA 46.9 kDa, and the expected band is marked with an asterisk. (TIF) [file pone.0116152.s001.tif]

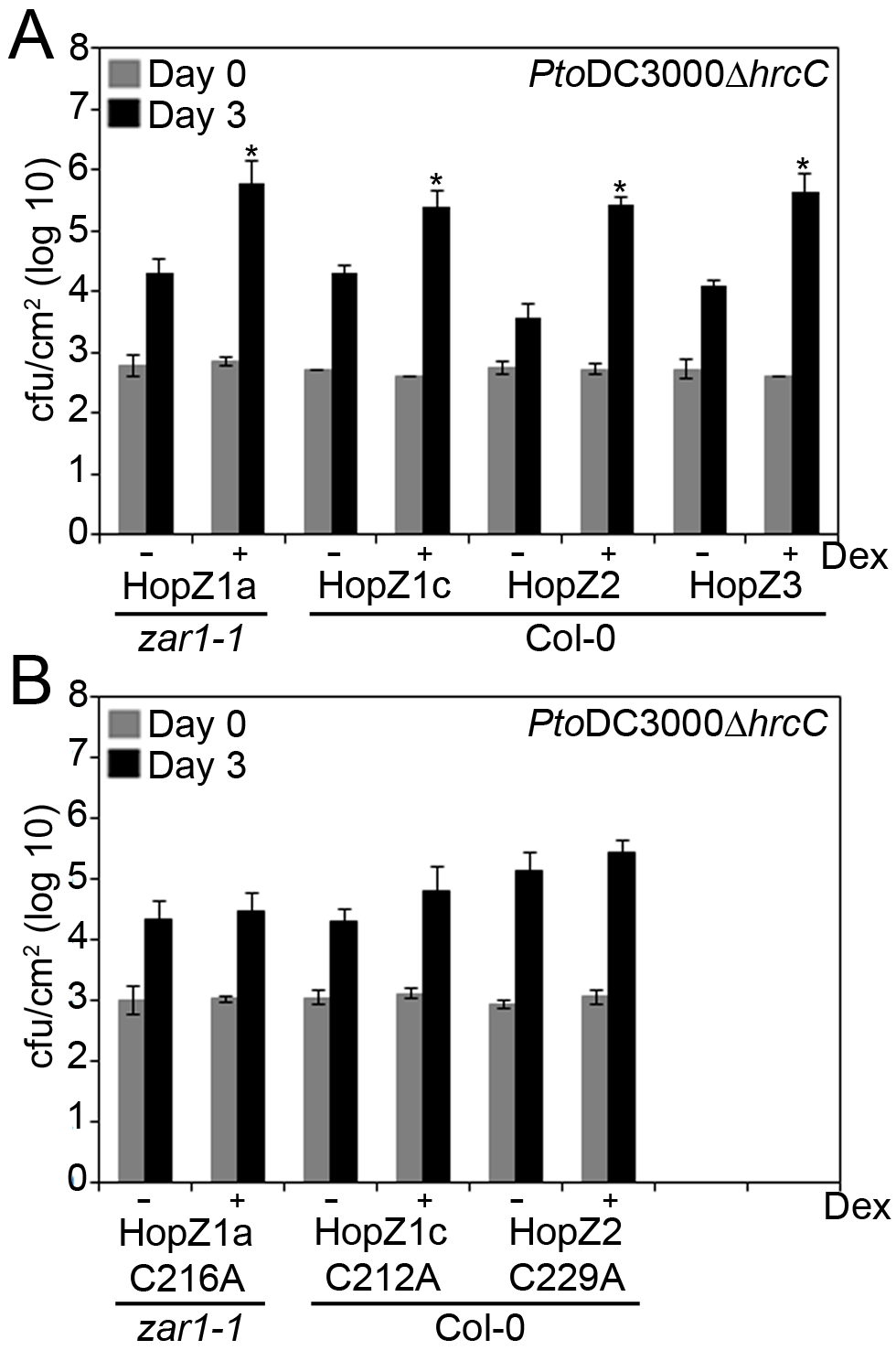

Supplement: S2 Fig — HopZ family members suppress PTI. (A) PtoDC3000ΔhrcC was pressure infiltrated at 1×10^5^ cfu/mL into transgenic HopZ1a in zar1-1 (line 14E), or HopZ1c (line 32A), HopZ2 (line 7G) or HopZ3 (line 36A) in Arabidopsis Col-0. Bacterial counts were determined one hour post-infection (Day 0) and 3 days post-infection (Day 3). Transgenic HopZ lines were sprayed with 30 µM dexamethasone or water 1 hour post-infiltration. Two-tailed homoschedastic t-tests were performed to test for significant differences. Within a plant genotype, dexamethasone-induced plants were compared to non-induced plants and significant differences are indicated by an asterisk (* P<0.01). Error bars indicate the standard deviation from the mean of 10 samples. Growth assays were performed at least 3 times. (B) Transgenic HopZ1aC/A (line 4G), and HopZ1cC/A (line 40F) lines were tested as in part A. Transgenic HopZ2C/A (line 5C) was sprayed 24 hours pre-infiltration as its expression level was lower than the other lines. We were unable to identify a second HopZ3C/A line that continued to express in the T3 generation. (TIF) [file pone.0116152.s002.tif]

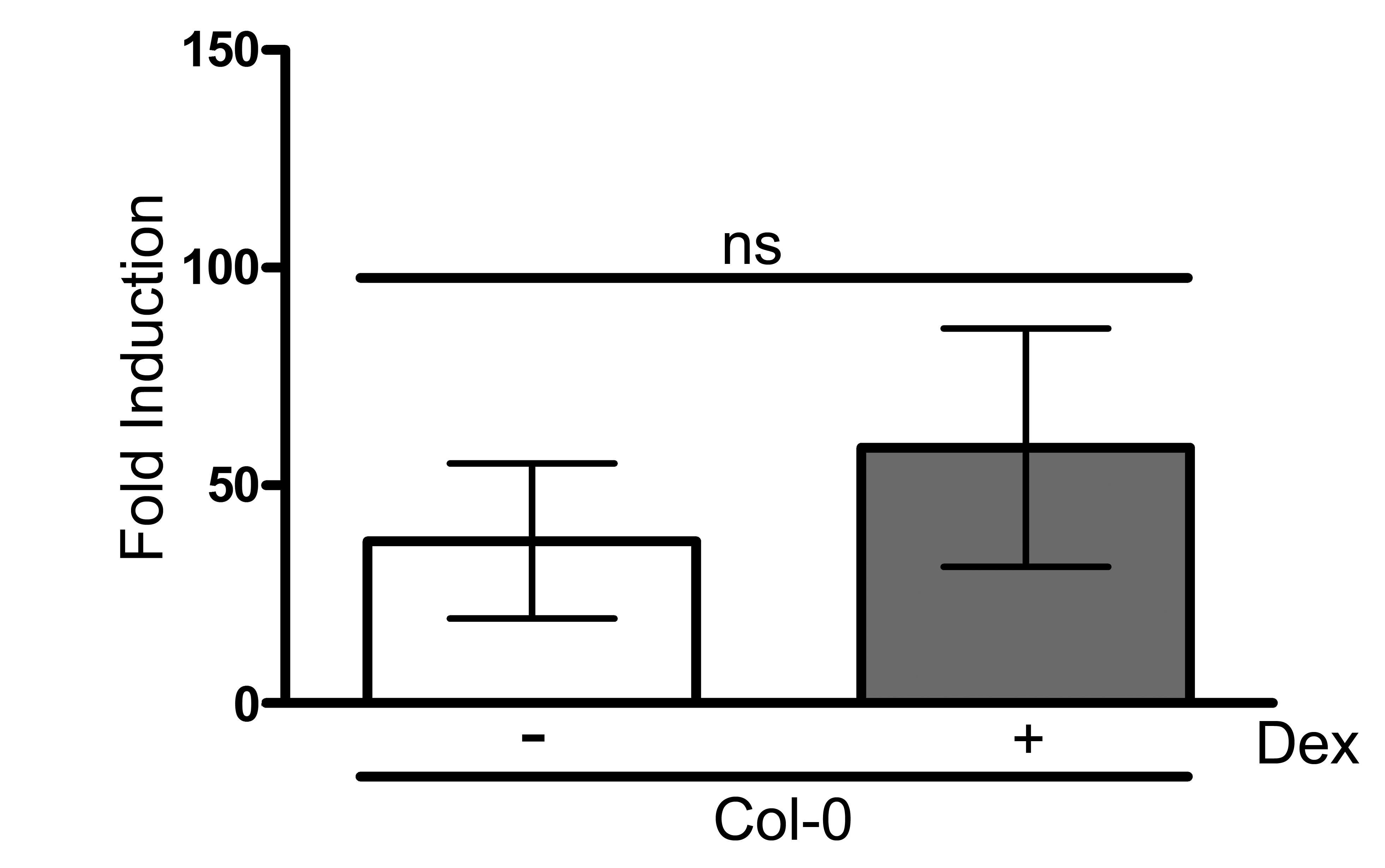

Supplement: S3 Fig — Dexamethasone does not affect ROS production in Arabidopsis Col-0 after flg22 induction. Untransformed Col-0 4 week-old plants were induced with 30 µM dexamethasone or mock treated with water 24 hours before sampling tissue. Tissue was treated with 2 µM flg22 44 hours after dexamethasone induction. ROS production was measured using a luminol-dependent chemiluminescence assay. Luminescence was measured for a total of 100 seconds over a 50 minute period from 3 plants per treatment. Each flg22-treated sample was normalized with the paired water treated sample to give a fold induction. Two-tailed homoschedastic t-tests were performed to test for significant differences. Within a plant genotype, dexamethasone-induced plants were compared to non-induced plants and no significant differences were observed (ns = not significant). Error bars indicate the standard deviation from the mean. Similar results were observed in two experiments. (TIF) [file pone.0116152.s003.tif]

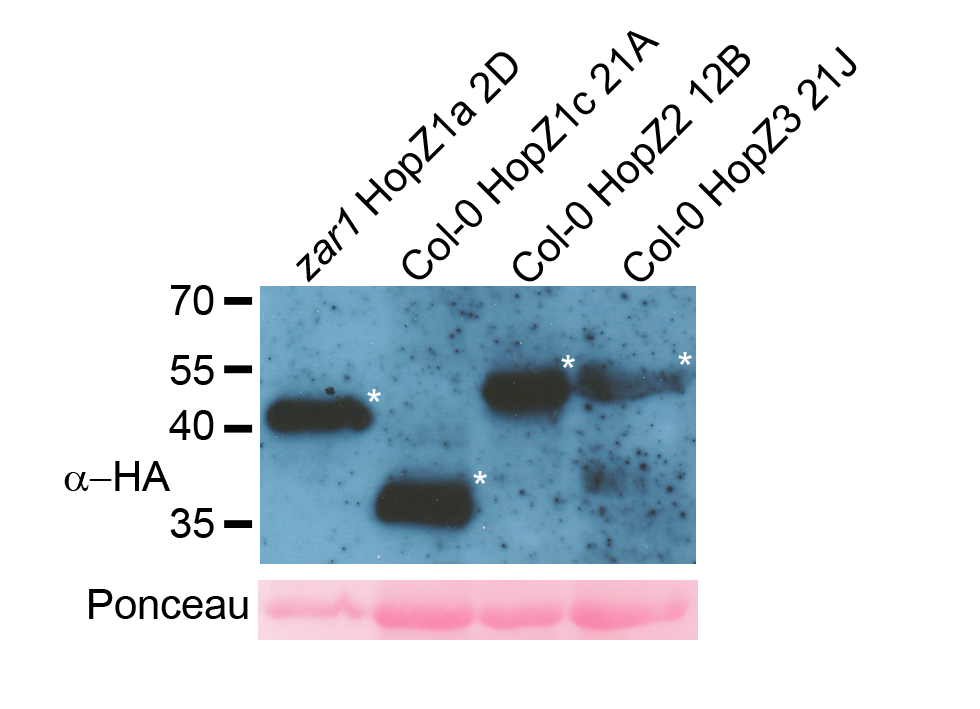

Supplement: S4 Fig — Transgenic HopZ family members cannot suppress ETI from related or unrelated T3SEs. Immunoblot analysis of HopZ proteins expressed in transgenic lines 8 hours after treatment with 30 µM dexamethasone or water. Transgenic HopZ1a is in a zar1-1 background while HopZ1c, HopZ2 and HopZ3 are in a Col-0 background. The Ponceau Red stained blot serves as the loading control. The expected sizes are as follows: HopZ1a-HA 42.1 kDa, HopZ1c-HA 30.5 kDa, HopZ2-HA 41.9 kDa, HopZ3-HA 46.9 kDa, and the expected band is marked with an asterisk. (TIF) [file pone.0116152.s004.tif]
