# Supplementary material for: Immunomodulation by the Pseudomonas syringae HopZ Type III Effector Family in Arabidopsis
Source: PLoS One. 2014 Dec 29;9(12):e116152. doi: 10.1371/journal.pone.0116152 (PMC4278861; doi:10.1371/journal.pone.0116152)
Supplement: S1 Table — GenBank accession numbers and P. syringae strain for each member of the HopZ family. (DOC) [file pone.0116152.s005.doc]

| **Table S1.** GenBank accession numbers and *P. syringae* strain for each member of the HopZ family | | |
| --- | --- | --- |
| **HopZ member** | ***P. syringae* Strain** | **GenBank accession** |
| HopZ1a | *P. syringae* pv. syringae A2 (*Psy*A2) | AAR02168 |
| HopZ1b | *P. syringae* pv. glycinea BR1 (*Pgy*BR1) | ABK13729 |
| HopZ1c | *P. syringae* pv. maculicola ES4326 (*Pma*ES4326) | AAL84243 |
| HopZ2 | *P. syringae* pv. pisi 895A (*Ppi*895A) | CAC16700 |
| HopZ3 | *P. syringae* pv. syringae B728a (*Psy*B728a) | AAF71492 |
